# Frequency and Characteristics of Metabolic Syndrome in Patients With Ischemic Stroke Admitted to a Tertiary Care Hospital in Karachi

**DOI:** 10.7759/cureus.9004

**Published:** 2020-07-05

**Authors:** Anum Amir, Mujtaba Hassan, Souhaib Alvi, Abdul Mueed, Sikander Idrees, Jibran Ashraf, Farhan Haleem, M Ali Khan

**Affiliations:** 1 Internal Medicine, Civil Hospital, Karachi, PAK; 2 Critical Care, National Institute of Cardiovascular Diseases, Karachi, PAK; 3 Medicine, Abbassi Shaheed Hospital, Karachi, PAK; 4 Cardiac Electrocardiography, National Institute of Cardiovascular Diseases, Karachi, PAK; 5 Neurosurgery, Sindh Government Hospital, Karachi, PAK; 6 Cardiology, National Institute of Cardiovascular Diseases, Karachi, PAK; 7 Gastroenterology, Jinnah Postgraduate Medical Centre, Karachi, PAK

**Keywords:** stroke, frequency, metabolic syndrome, characteristics

## Abstract

Introduction

Metabolic syndrome (MetS) is defined as a syndrome of truncal obesity, insulin resistance, hypertension, hypertriglyceridemia, and dyslipidemia. It is well known that MetS increases the risk of cardiovascular disease and adverse events. Each of its components is associated with an increased risk of cardiovascular disease, but data on the association with ischemic stroke are scarce. At the international level, a significant body of research has been conducted on this issue, but the situation is very different in Pakistan. Very little data are present on the subject matter. This study is an endeavor in this direction, generating data, that can be used in early identification and developing treatment services for patients with ischemic stroke having MetS.

Aims

To determine the frequency of MetS in ischemic stroke patients admitted to a tertiary care hospital in Karachi, Pakistan.

Methods

This six-month observational and cross-sectional study was conducted at Medical Unit I, Jinnah Postgraduate Medical Centre from July 1, 2019, to December 31, 2019. Patients with a diagnosis of acute ischemic stroke were enrolled. Detailed history, physical examination, and biochemical measurements were noted. The presence of MetS was defined in accordance with the National Cholesterol Education Program (NCEP) Adult Treatment Panel III (ATP III)/American Heart Association (AHA) guidelines.

Results

A total of 224 patients fulfilling the inclusion criteria were inducted into this study. The mean age of presentation was 61.04 ± 14.72 years, and more than two-thirds of the patients were ≥60 years of age. A total of 150 (66.96%) patients with ischemic stroke also had MetS. The male-to-female ratio in this group was 2:1. The most common variables constituting the MetS were truncal obesity, hypertension, and dyslipidemia. The median MetS score was 3.

Conclusions

MetS is highly prevalent in patients presenting with ischemic stroke irrespective of age or gender. The three most deranged and common components of MetS in these patients are truncal obesity, hypertension, and dyslipidemia.

## Introduction

Stroke is one of the leading causes of chronic disability and death worldwide, and its incidence progressively increases with age. In Pakistan, the estimated stroke incidence is close to 250 per 100,000 population, which means that there are 350,000 new stroke patients every year in the country [1]. A recent community-based survey suggested an estimated 21.8% prevalence of stroke in an urban slum of Karachi, Pakistan. Stroke-specific fatality has been reported between 7% and 20% in studies from Pakistan [2], with similar studies from China reporting mortality rates between 3% to 25% [2]. The overall risk of recurrent stroke, fatal or nonfatal, is about 20% at five years [3].

The metabolic syndrome (MetS) is defined as a syndrome of truncal obesity, insulin resistance, hypertension, hypertriglyceridemia, and dyslipidemia. The prevalence of MetS has been increasing at an alarming rate throughout the world. In the United States, the current prevalence is estimated to be at 27% [4]; in Europe, it is 15.7% in men and 14.2% in women [5]; and in China, it is 13.7% [6]. The presence of MetS is strongly associated with the development of hyperuricemia, diabetes, hypertension, cardiovascular disease, and all-cause mortality [7].

MetS increases cardiovascular risk, and each of its components is associated with an increased risk of cardiovascular disease [8], but data on the association with ischemic stroke are scarce. One study has shown that about 62% of ischemic stroke patients had MetS [9]. In a cross-sectional study from the Third National Health and Nutrition Examination survey, MetS was significantly associated with self-reported myocardial infarction and stroke [10]. The presence of the MetS was associated with increased cerebrovascular mortality [11]. In a prospective study of patients with hypertension without cardiovascular diseases, MetS was an independent predictor for both cardiovascular and cerebrovascular disease [12].

## Materials and methods

Operational definitions

Acute ischemic stroke is defined as focal neurological deficit (weakness of the right or left side of the body) lasting for >24 hours with evidence of hypodensity on CT scan of the brain.

MetS was diagnosed according to the National Cholesterol Education Program (NCEP) Adult Treatment Panel III(ATP III)/American Heart Association (AHA) guidelines. MetS was diagnosed if a patient had ≥3 of the following criteria: serum triglyceride levels ≥ 150 mg/dL, serum high density lipid cholesterol (HDL-C) levels < 40 mg/dL for males and <50 mg/dL for females, systolic blood pressure (SBP) ≥ 130 mmHg or diastolic blood pressure (DBP) ≥ 85 mmHg, fasting blood glucose (FBS/FBG) ≥ 110 mg/dL, and waist circumference > 85 cm in females and > 90 cm in males (truncal obesity).

Methodology

This was a six-month observational cross-sectional study conducted at Medical Unit I, Jinnah Post Graduate Medical Center, Karachi, from July 1, 2019, to December 31, 2019. Non-probability consecutive sampling technique was used.

Inclusion Criteria

Patients >18 and <70 years of age of either gender with ischemic stroke as per operational definition was included.

Exclusion Criteria

Patients with hemorrhagic stroke determined by hyperdensity on CT scan of the brain, ischemic stroke of <24 hours’ duration, renal failure determined by serum creatinine of >2 mg/dL, septicemia, multiple end-stage organ failure, and terminal malignancy were excluded.

Statistical analysis

A database was developed using SPSS version 21 (IBM Corp., Armonk, NY, USA). Mean and standard deviation (SD) were calculated for age, waist circumference (truncal obesity), SBP, DBP, FBS, serum triglycerides, and serum HDL-C. Frequency and percentages was calculated for age, gender, and MetS and its components. Different components of the MetS were also presented as mean with SD. Effect modifiers were controlled through stratification of age, gender, truncal obesity, hypertension, glucose intolerance, hypertriglyceridemia, and dyslipidemia to assess the effect of these on outcome variables applying the chi-square test. A p-value of <0.05 was considered as significant.

## Results

Demographics

A total of 224 patients were admitted to the ward with a confirmed diagnosis of acute ischemic stroke during the study period. The frequency of MetS in these patients was 66.94%. The female-to-male ratio in this study was 1:2.11, and in patients with MetS syndrome, this ratio was 1:2. The mean age of presentation was 61.04 ± 14.72 years. These results are summarized in Table 1. No single demographic variable was found to have statistical significance.

**Table 1:** Demographics of patients with and without metabolic syndrome.

Parameter	N = 224 (%)		
	Metabolic Syndrome Present	Metabolic Syndrome Absent	p-Value
Male	100 (44.64%)	52 (23.21%)	>0.05
Female	50 (22.32%)	22 (9.82%)	>0.05
60 ≥ years of age	92 (41.10%)	44 (19.64%)	>0.05
60 ≤ years of age	58 (25.89%)	30 (13.39%)	>0.05
Total	150 (66.96%)	74 (33.03%)	<0.05
Age (mean)	64.40 ± 8.72 years	68.81 ± 22.72 years	>0.05
Overall age (mean)	61.04 ± 14.72 years		

Risk factors/components of MetS and their frequency​​​​​​​

The median MetS score was 3 out of 5. The most prevalent deranged risk factor for MetS was truncal obesity; a whopping 86% of the patients had increased waist circumference and that too by some margin. Almost all patients had raised SBP and dyslipidemia (low HDL-C). This was surprising as a majority of the patients were using anti-hypertensive and lipid-lowering (and HDL-C raising) drugs. The three most deranged parameters were also the most common ones, i.e., truncal obesity, hypertension (systolic), and dyslipidemia. This was also the most common trifecta of risk factors comprising the MetS in this study.

Truncal obesity, hypertension (systolic), and dyslipidemia were found to be clinically and statistically significant within the MetS group. However, overall, in this study, no single factor was found to be statistically significant (see subgroup analysis). Characteristics of MetS are given in Table 2. Frequency of the risk factors is summarized in Table 3.

**Table 2:** Risk factors/components of the metabolic syndrome.

Parameter	Mean ± standard deviation
Waist (inches)	41.04 ± 14.725
Systolic blood pressure (mmHg)	139.29 ± 24.50
Diastolic blood pressure (mmHg)	84.33 ± 10.707
Fasting blood sugar (mg/dL)	102.97 ± 23.85
Serum triglycerides (mg/dL)	143.58 ± 33.77
High density lipid - cholesterol (mg/dL)	23.20 ± 4.038
Median metabolic syndrome score (out of 5)	3

**Table 3:** Frequency of risk factors/components in patients with metabolic syndrome. *p-Values are given for within the metabolic syndrome group.

Risk Factor	N=150	p-Value*
Increased waist circumference	129 (86.0%)	<0.05
Raised systolic/diastolic blood pressure	124 (82.66%)	<0.05
Dyslipidemia	98(65.33%)	<0.05
Hypertriglyceridemia	34 (22.66%)	>0.05
Pre-diabetes/glucose intolerance	20 (13.33%)	>0.05

## Discussion

The presence of MetS significantly increases the incidence of ischemic stroke in patients; the incidence of stroke is slightly higher for males [13], similar to our results. Data for Caucasian patients show comparable trends [14]. Long-term follow-up results by Zhang et al. from China demonstrated nearly identical tendencies [15]; this study additionally identified truncal obesity and hypertension as independent risk factors for ischemic stroke. While local extensive data are scarce, the results and outcomes of our study correspond well with data from the world over.

In light of previous data, it is easy to see that certain components, namely truncal obesity and hypertension, carry an increase risk (or hazard ratio) of adverse outcomes [16]. Our results correlate well with these findings. More than 80% of our patients had either one or both of these components present in the MetS group. The frequency of these components was high even in the non-MetS group, making them independent risk factors for incidence of ischemic stroke and outcomes, as discussed above.

Hachinski et al. [17] described the effects of increased lipid levels on outcomes of ischemic stroke. Only a small percentage of our patients had hypertriglyceridemia with MetS; this percentage was even lower in the non-MetS group. Even the mean for triglyceride levels was below the desired/optimal levels. On the other hand, dyslipidemia (low HDL-C levels) was far more prevalent. In the MetS group, 65.33% of the patients had it.

Dyslipidemia and not hypertriglyceridemia as the major cholesterol pathology is hard to explain, especially in the context of statin use and ischemic stroke. We could not ascertain as to why so many of the patients who were already on statin had reduced HDL-C levels. The mean even with SD is 20 points below the desired level. Statins increase HDL-C levels [18], why was this not the case in these patients? Was it non-compliance or drug tolerance? Was it due to other co-morbids and sedentary lifestyle? Or does MetS reduce the efficacy of statins? Perhaps, a more detailed analysis at another time could answer this question; for the moment this query evaded us (the frequency of dyslipidemia and use of statin were far too prevalent for this to be an error of sample size).

The frequency of glucose intolerance or prediabetes was exceptionally low in our study; in fact, it was the least frequent component in both the MetS and non-MetS groups. The relationship between MetS and glucose intolerance is well established, but MetS without prediabetes is also not uncommon [19]. Type II diabetes is also an independent risk factor for ischemic stroke [20]. Thus, the expectation was to find a large number of patients with prediabetes/glucose intolerance or at the very least glucose tolerance to be a major component of the MetS in the study. Yet, this did not happen.

The authors are of the opinion that FBS/FBG, while a reasonable variable for calculating MetS, is an ineffective tool for determining glucose intolerance. FBS can be influenced by a number of factors such as age, alcoholism, certain fruit intake, meat or fish intake, occupation, waist circumference, family history of diabetes, elderly female, improper fasting period, and lab error [21]. To rule out every single one of these variables in every patient would be time-consuming and cumbersome. As such, it would be pertinent to calculate glycemic status through more reliable tests such glycosylated hemoglobin A1c (HbA1c) or HOMA-IR (Homeostatic Model Assessment of Insulin Resistance) in the future.

Shortcomings

This study was limited by a number of factors. Most importantly, it did not cover the cardiovascular risk factors associated with ischemic stroke. Etiologies such as atrial fibrillation, atrial flutter, coronary artery disease, carotid artery stenosis, prothrombotic states, and history of stent placement with use of anticoagulants were neither recorded nor evaluated. Almost all patients were using statins before presentation, and the effects and correlations of statin use, compliance, and dosage were not evaluated. Nutritional assessment was not performed. Use of other drugs, smoking, substance abuse, surgical history, and use of quack medications were not recorded. While infectious disease panel was carried out, it was not analyzed for the purposes of this study. Mortality rates and eventual outcomes were not analyzed.

## Conclusions

Frequency of MetS in patients with ischemic stroke is high; more than two-thirds of the patients with ischemic stroke have MetS. Both genders are affected by this without bias and irrespective of age. Truncal obesity, hypertension, and dyslipidemia are the most common components of MetS in these patients.
